# Incidence and outcome of paediatric cardioembolic stroke: a nationwide population-based study

**DOI:** 10.1093/esj/aakag070

**Published:** 2026-06-29

**Authors:** Sarah Juul, Thomas Clement Truelsen, Alexander Bastian Koldborg, Christina Engel Hoei-Hansen, Julie Brix Bindslev

**Affiliations:** Department of Neonatology, University Hospital of Copenhagen, Rigshospitalet, Copenhagen, Denmark; Department of Paediatrics, University Hospital of Copenhagen, Rigshospitalet, Copenhagen, Denmark; Department of Clinical Medicine, University of Copenhagen, Copenhagen, Denmark; Department of Neurology, University of Copenhagen, Rigshospitalet, Copenhagen, Denmark; Department of Neurology, University of Copenhagen, Rigshospitalet, Copenhagen, Denmark; Department of Paediatrics, University Hospital of Copenhagen, Rigshospitalet, Copenhagen, Denmark; Department of Clinical Medicine, University of Copenhagen, Copenhagen, Denmark; Department of Neurology, University of Copenhagen, Rigshospitalet, Copenhagen, Denmark

**Keywords:** arterial ischaemic stroke, cardiac disease, cardioembolic stroke, outcome, paediatric stroke

## Abstract

**Introduction:**

Cardiac disease is a leading cause of arterial ischaemic stroke (AIS) in children, yet data on paediatric cardioembolic stroke remain limited. This study investigated the incidence rate, underlying cardiac diseases and outcome of paediatric cardioembolic stroke in Denmark.

**Patients and methods:**

In a nationwide registry, we identified all children with a stroke or stroke-related diagnosis (2013–2020). Arterial ischaemic stroke events were confirmed by medical record review and classified using the Childhood AIS Standardized Classification and Diagnostic Evaluation (CASCADE) criteria as definite cardioembolic, probable cardioembolic or non-cardioembolic. Neuroimaging and neurological outcomes were extracted from medical records. Follow-up time was 2 years.

**Results:**

Cardioembolic stroke was identified in 31 (24.6%) of 126 children with AIS, corresponding to an incidence rate of 0.28 (95% CI, 0.20–0.40) per 100,000 person-years. Congenital heart diseases were the most common underlying conditions (*n* = 26, 83.9%). Among children undergoing angiography, LVOs were present in 41.2% of the cardioembolic group and in 18.5% of the non-cardioembolic group (*P*-value .060). The frequency of neurological impairments was similar between cardioembolic and non-cardioembolic AIS (48.4% vs 43.6%, *P* = .681). When limiting analysis to definite cardioembolic stroke, neurological impairment was more common in the cardioembolic than the non-cardioembolic group (73.3% vs 43.6%, *P* = .050), though this association was non-significant after adjusting for stroke severity.

**Discussion:**

Cardioembolic stroke accounted for nearly one-quarter of paediatric AIS events. While clinical outcomes were similar between cardioembolic and non-cardioembolic AIS, definite cardioembolic stroke was associated with a higher risk of incomplete neurological recovery, highlighting the need for improved preventive strategies.

## Graphical Abstract

**Graphical Abstract:**
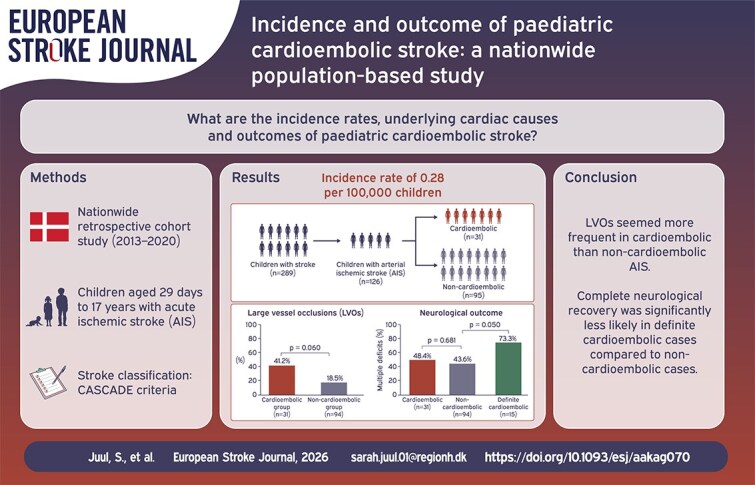

## Introduction

Paediatric stroke is a rare diagnosis, however, according to international data, it is classified among the 10 leading causes of childhood mortality.^1^ In a systematic review from Australia,^2^ the reported incidence rate of paediatric ischaemic strokes varies from 0.9 to 7.9 per 100,000 person-years, with a pooled incidence rate for arterial ischaemic stroke (AIS) of 1.28 (95% CI, 0.75–2.19) per 100,000 person-years. Despite its relatively low incidence, paediatric stroke is associated with substantial long-term morbidity. It is estimated that between 50% and 87% of affected children experience long-term neurological sequelae, encompassing physical, cognitive and psychosocial impairments.^3–5^ Given its significant impact on the quality of life of both the child and their family, these findings underscore the clinical severity of paediatric stroke and highlight the importance of improving the identification of associated risk factors.^6^

Arterial ischaemic stroke is the most common stroke subtype in children and is frequently caused by thromboembolic events, with cardiac disease representing one of the most important risk factors.^7^

In the United States, it’s estimated that approximately 1.3% of children are diagnosed with cardiac disease.^8^ This encompasses a wide spectrum of cardiac disorders, ranging from asymptomatic conditions to complex cyanotic congenital heart disease (CHD) requiring early surgical intervention. It is well established that children with underlying heart conditions are at increased risk of developing AIS, with studies reporting an association between cardiac disease and AIS in approximately 10%–30% of paediatric cases.^3^^,^^9^^,^^10^ Children with cardiac disease often exhibit altered cerebral and systemic blood flow due to shunting and haemodynamic instability, all of which increase the risk of embolic thrombus formation.^11^

In adults, cardioembolic stroke has been shown to be associated with more severe stroke outcomes than other AIS causes.^12^ Similarly, a multicentre retrospective cohort study demonstrated a higher mortality risk in children with AIS related to cardiac diseases compared to other AIS causes.^13^ However, data on the association between cardio-embolism and stroke outcome in children are scarce, and the International Paediatric Stroke Study Group has emphasised the need for further research on paediatric cardioembolic stroke.^14^ Clarifying the relation between cardio-embolism and AIS outcome may improve the understanding of the burden of paediatric cardiac disease and help improving preventive strategies in children with cardiac disease.

Hence, the objective of this nationwide study was to investigate the incidence rate, underlying cardiac diseases and clinical outcomes in children with cardioembolic stroke in Denmark.

## Patients and methods

This nationwide retrospective population-based study was conducted in Denmark within the setting of a public healthcare system. All Danish residents are assigned a 10-digit identification number at birth or upon immigration, which enables accurate linkage of data across various healthcare registries and between registries and medical records. The study methods and data were reported in accordance with the Strengthening the Reporting of Observational Studies in Epidemiology (STROBE) guidelines for cohort studies. Registries used in this study included (1) the Danish National Register of Patients (DNRP), a comprehensive nationwide registry that contains data on all discharges from non-psychiatric Danish hospitals since 1970, including information on admission and discharge dates, as well as discharge diagnosis codes defined by the International Classification of Diseases, 10th edition (ICD-10); (2) the Civil Registration System which holds information on vital status for Danish residents, including time of death or emigration.

### Study population

Study participants were identified by a search in DNRP using the following ICD-10 Codes: I60 (subarachnoid haemorrhage, SAH); I61–62 (intracerebral haemorrhage, ICH); I63 excl I63.6 (ischaemic stroke, AIS); I64 (unspecified stroke); I63.6, I67.6, I67.6A and DG08 (central venous thrombosis); DG45 (TIA) and I67–68 excl I67.6 and I67.6A (other cerebrovascular diseases).

For all identified children, medical records were retrieved for validation of possible stroke events. The validation was conducted in accordance with the stroke definition established by the American Heart Association/American Stroke Association.^15^ Arterial ischaemic stroke was defined as cerebral cell death due to ischaemia, confirmed by imaging or other objective evidence of ischaemic injury, or by persistent clinical symptoms (>24 h or until death), with other causes excluded.

Children aged 29 days to 17 years with confirmed first-ever AIS in the period January 2013–December 2020 were included. Neonatal cases were not included due to many underlying and co-existing pathophysiological differences.

### Cardioembolic stroke

Among children with confirmed AIS, we identified those with cardioembolic stroke. Cardioembolic stroke was defined in accordance with the Childhood AIS Standardized Classification and Diagnostic Evaluation (CASCADE) criteria, modified from Bernard et al.^16^ The children were classified as: (1) definite cardioembolic stroke; (2) probable cardioembolic stroke or (b) non-cardioembolic.

The category of “definite cardioembolic stroke” encompassed children with a high-risk cardiac source for cerebral embolism (eg, CHD with abnormal cardiac function, arrhythmia, endocarditis), or a cardiac procedure within 30 days prior to stroke and involvement of a large- or medium-sized cerebral artery or > 1 arterial territory.^16^ The category of “probable cardioembolic stroke” was used for children with involvement of ≥ 1 arterial territory and one of the following: (1) patent foramen ovale (PFO) or (2) other subtle cardiac anomaly.

Cardiac procedures were categorised as cardiac surgery, cardiac catheterisation or extracorporeal membrane oxygenation. Time from cardiac procedure to stroke onset was obtained from medical records and categorised as 0–24 h, 25–72 h, 3–14 days, 15 days–3 months, 3 months–1 year or 1–5 years.

### Outcome

The frequency and severity of neurological disability were assessed in accordance with the paediatric stroke outcome measure (PSOM). The PSOM is a systematic neurological assessment including 5 subscales (sensorimotor right, sensorimotor left, language production, language comprehension and cognitive/behavioural). Each subscale is scored from 0 to 2. In this study, we retrospectively scored the PSOM based on prior evaluations by paediatricians, neurologists or neuropsychologists. In children with more than one assessment, we used the assessment conducted closest to 2 years after the stroke. In accordance with Slim et al.,^17^ the overall neurological outcome was classified as follows: normal = 0–0.5 in all PSOM subscales; mild = 1 in 1 subscale and < 1 in the remaining subscales; moderate = 1 in ≥ 2 subscales OR 2 in only 1 subscale and < 1 in all remaining subscales; severe = 2 in only 1 subscale and > 1 in any of remaining subscales OR 2 in > 2 subscales.

### Statistical analysis

Using Poisson regression analysis, we estimated the incidence rate of paediatric cardioembolic stroke per 100,000 person-years with 95% CIs. The incidence rate was calculated by dividing the number of children with cardioembolic stroke by the number of person-years at risk. Person-time at risk data were retrieved from the Civil Registration System. The children were censored from the population at risk at the time of stroke, death or emigration.

Baseline PedNIHSS scores were compared between the cardioembolic and non-cardioembolic groups using the Mann–Whitney *U* test. Binary logistic regression analysis was used to examine the association between PedNIHSS score, cardioembolic stroke, definite cardioembolic stroke and clinical outcome. Results were reported as odds ratios (ORs) with 95% CIs.

Variables were summarised descriptively according to stroke aetiology (cardioembolic vs non-cardioembolic). The frequency of neurological deficits and PSOM scores was compared between groups using Fisher’s exact test. Complete case analysis was applied to account for missing data. A 2-sided *P*-value ≤ .05 was considered statistically significant.

All statistical analyses were performed using R statistical software version 4.2.282 and SPSS version 28.0.0.08.

### Ethics

The study was approved by the Danish Data Protection Agency (J. no.: P-2020-451 and J. no.: P-2021-539) and the Centre for Regional Development, Health Research and Innovation, the Capital Region of Denmark (J. no.: R-21048871).

## Results

**Figure 1:**
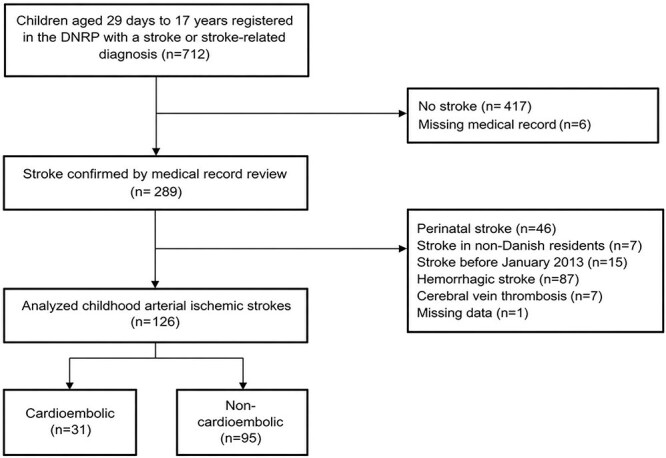
Cohort flow diagram. Cohort diagram with numbers of patients belonging to each group. The diagram illustrates the identification of children from the Danish National Register of Patients and the subsequent stepwise exclusions to reach the final cohort of children with acute ischaemic stroke. Abbreviation: DNRP = Danish National Register of Patients.

Between January 2013 and December 2020, a total of 712 children were registered in the DNRP with a stroke or a stroke-related diagnosis. Following a detailed review of medical records, 417 patients were excluded due to an incorrect stroke diagnosis, and an additional 6 patients for missing medical records. Stroke was confirmed in 289 patients. Subsequently, the following patient groups were excluded (numbers in brackets): perinatal stroke (*n* = 46), stroke in non-Danish residents (*n* = 7), stroke occurring before January 2013 (*n* = 15), haemorrhagic stroke (*n* = 87) and cerebral venous thrombosis (*n* = 7). Ultimately, 127 patients with AIS were identified and information on possible AIS aetiologies was obtained in 126 of these (Figure 1).

Echocardiography was performed in 101 (80.2%) of 126 children and was transthoracic in 56 (55.4%), transesophageal in 9 (8.9%) and unspecified in 39 (38.6%). Electrocardiography was performed in 82 of 126 (65.1%) children, whereas Holter monitoring or event recording was performed in 9 (7.1%).

Cardioembolic stroke was present in 31 of 126 (24.6%) children, including 15 children with definite cardioembolic stroke and 16 with probable cardioembolic stroke. The overall incidence rate of first-ever paediatric cardioembolic stroke was 0.28 (95% CI, 0.20–0.40) per 100,000 person-years.

### Demographic and clinical characteristics

The median age at presentation was 13 years (IQR: 5–16) in the cardioembolic group and 6 years (IQR: 3–13) in the non-cardioembolic group (*P* = .030). In both groups, AIS was more frequent in males, with males accounting for 58.1% in the cardioembolic group and 60.0% in the non-cardioembolic group.

Information on PedNIHSS was obtained in 112 of 126 children with AIS. The median PedNIHSS score was 3 (IQR 0–8) in the cardioembolic group and 2 (IQR 0–5) in the non-cardioembolic group (*P* = .416). For children classified as having definite cardioembolic AIS, the median PedNIHSS was 3.5 (IQR 0–8.5) with no significant difference compared to the non-cardioembolic group (*P*-value .474).

A cardiac disease was known prior to stroke in 14 of the 31 children (45.2%) with cardioembolic stroke, while diagnosed after the stroke in 17 (54.8%). Among the 14 children with a pre-existing cardiac diagnosis, 11 were classified in the definite cardioembolic group and 3 in the probable cardioembolic group. The 3 children classified in the probable cardioembolic group had a PFO.

Among children with cardioembolic stroke, a total of 15 patients (48.4%) were diagnosed with severe symptomatic heart diseases, including: atrioventricular septal defect, double outlet right ventricle/transposition of the great arteries, tricuspid atresia, single ventricle physiology, Ebstein anomaly, coarctation of the aorta, aortic stenosis, ventricular septal defect (VSD) and pulmonary hypertension (PH). The most common cardiac abnormalities were PFO or atrial septal defect observed in 16 patients (51.6%) (Table 1).

**Table 1:** Cardiac diseases in children with definite and probable cardioembolic arterial ischaemic stroke.

	*n*	%
**Definite cardioembolic stroke**	15	48.4
***CHD***	10	32.3
** AVSD**	3	9.7
** DORV**	1	3.2
** DORV with TGAs**	1	3.2
** Tricuspid atresia with single ventricle physiology**	1	3.2
** Aortic stenosis (AS)**	1	3.2
** Ebstein anomaly**	1	3.2
** Coarctation of the aorta with AS**	1	3.2
** VSD with PH**	1	3.2
***Acquired cardiac disease***	5	16.1
** Arrhythmia**	2	6.5
** Cardiac myxoma**	1	3.2
** Mural thrombosis**	1	3.2
** Mechanical heart valve**	1	3.2
**Probable cardioembolic stroke**	16	51.6
** Patent foramen ovale/atrial septal defect (PFO/ASD)**	16	51.6

Abbreviations: AS: aortic stenosis; ASD, atrial septal defect; AVSD, atrioventricular septal defect; CHD, congenital heart disease; DORV, double outlet right ventricle; PFO, patent foramen ovale; PH, pulmonary hypertension; TGAs, transposition of the great arteries; VSD, ventricular septal defect.

The most frequently acquired cardiac condition was arrhythmia, observed in 2 patients (6.5%). One child had a third-degree (grade 3) atrioventricular block and one had atrial standstill. Other acquired diagnoses included mural thrombosis associated with myocarditis, one case of cardiac myxoma and one case of mechanical heart valve.

### Procedure and anti-coagulation therapy

Among children with a cardiac disease known prior to stroke, 3 of 14 were treated with antithrombotic therapy at the time of stroke; 1 was treated with acetylsalicylic acid 75 mg daily, 1 with warfarin 2.5 mg daily (above therapeutic level) and 1 with warfarin 1.25–2.5 mg daily (within therapeutic level).

Nine children underwent cardiac surgery, of which 6 underwent sternotomy and open surgery, and 3 children underwent catheter device operations. Among the 6 children undergoing open surgery, stroke was recognised within 3 days of surgery in 2 and within 14 days in 3. Four children developed AIS 1–5 years after surgery of which 3 underwent catheterisation. Four children were on preventive antithrombotic treatment prior to surgery. None of the children undergoing cardiac surgery underwent pre-surgery screening for intracardiac thrombi.

### Neuroimaging

Neuroimaging data were available for 125 of 126 children with AIS. Multiple infarctions were observed in 10 of 31 (32.3%) children with cardioembolic stroke and in 26 of 94 (27.7%) children with non-cardioembolic stroke, with no significant difference between groups (*P* = .651). Most children had infarcts in the anterior circulation (cardioembolic 67.7%; non-cardioembolic 56.4%; *P* = .298) (Table 2).

**Table 2:** Imaging characteristics in children with non-cardioembolic and cardioembolic stroke.

	Cardioembolic (*n* = 31)	Non-cardio-embolic (*n* = 94) ^a^	*P*-value ^b^	Definite cardio-embolic (*n* = 15)	*P*-value ^c^
**Initial neuroimaging**					
**CT**	14 (45.1)	42 (44.7)	1	7 (46.7)	1
**CT with CTA**	3 (9.7)	6 (6.4)	0.689	1 (6.7)	1
**MRI**	8 (25.8)	28 (29.8)	0.82	3 (20.0)	0.548
**MR with MRA**	3 (9.7)	12 (12.8)	0.76	2 (13.3)	1
**NR**	3 (9.7)	6 (6.4)	0.689	2 (13.3)	0.303
**Number of infarcts**					
**Single**	21 (67.7)	60 (63.8)	0.829	9 (60.0)	0.78
**Multiple**	10 (32.3)	26 (27.7)	0.651	6 (40.0)	0.366
**No infarcts**	0	8 (8.5)	0.199	0	0.596
**Infarct location**					
**Anterior**	21 (67.7)	53 (56.4)	0.298	10 (66.7)	0.578
** Frontal lobe**	11 (35.5)	17 (18.1)	0.051	6 (40.0)	0.083
** Parietal lobe**	12 (38.7)	16 (17.0)	0.023	5 (33.3)	0.161
** Temporal lobe**	4 (12.9)	9 (9.6)	0.735	1 (6.7)	1
** Basal ganglia**	9 (29.0)	34 (36.2)	0.52	4 (26.7)	0.569
** Internal capsule**	5 (16.1)	16 (17.0)	1	1 (6.7)	0.458
** Corona radiata**	5 (16.1)	14 (14.9)	1	3 (20.0)	0.701
** Retina**	0	1 (1.1)	1	0	1
** Other**	0	3 (3.2)	0.574	0	1
**Posterior**	13 (41.9)	34 (36.2)	0.67	7 (46.7)	0.567
** Thalamus**	5 (16.1)	12 (12.8)	0.763	2 (13.3)	1
** Brainstem**	0	10 (10.6)	0.066	0	0.352
** Occipital lobe**	6 (19.4)	10 (10.6)	0.223	3 (20.0)	0.384
** Cerebellum**	3 (9.7)	10 (10.6)	1	2 (13.3)	0.67
** Other**	1 (3.2)	2 (2.1)	1	0	1
**Angiographic findings**					
**Angiography performed**	17 (54.8)	65 (69.1)	0.191	6 (40.0)	0.04
**LVOs^d^**					
** Any**	7 (41.2)	12 (18.5)	0.06	4 (66.7)	0.02
** Anterior circulation**	6 (35.3)	7 (10.8)	0.023	3 (50.0)	0.14
** ICA**	0	2 (3.1)	1	0	1
** ICA and MCA**	0	2 (3.1)	1	0	1
** MCA**	6 (35.3)	3 (4.6)	0.002	3 (50.0)	0.033
** ACA**	0	0	–	0	–
** Posterior circulation**	1 (5.9)	5 (7.7)	1	1 (16.7)	1
** BA**	0	4 (6.2)	0.575	0	1
** PCA**	0	1 (1.5)	1	0	1
** Intracranial VA**	0	0	–	0	–

Abbreviations: ACA, anterior cerebral artery; AIS, arterial ischaemic stroke; BA, basilar artery; ICA, internal carotid artery; MCA, middle cerebral artery; NR, not reported; PCA, posterior cerebral artery; VA, vertebral artery.

^a^Information on neuroimaging obtained in 125 of 126 children with AIS.

^b^Fisher’s exact test: cardioembolic vs non-cardioembolic.

^c^Fisher’s exact test: definite cardioembolic vs non-cardioembolic.

^d^Data on LVOs reported for children who underwent angiography (CTA, MRA or digital subtraction angiography) during admission for stroke.

Angiography was performed in 82 children with AIS, including 17 of 31 (54.8%) with cardioembolic stroke and 65 of 94 (69.1%) with non-cardioembolic stroke. Among children undergoing angiography, LVOs were present in 7 of 17 (41.2%) in the cardioembolic group and in 12 of 65 (18.5%) in the non-cardioembolic group (*P*-value .060) (Table 2).

### Clinical outcome

Information on clinical outcome was obtained in 125 of 126 children with AIS. Median time from AIS to follow-up was 22.4 (IQR 13.3–28.6) months.

Neurological impairments occurred in 48.4% of children with cardioembolic stroke and 43.6% with non-cardioembolic stroke (*P* = .681) (Table 3). The most common neurological deficits were sensorimotor deficits (cardioembolic 41.9%; non-cardioembolic 46.8%) and cognitive and behavioural deficits (cardioembolic 25.8%; non-cardioembolic 24.5%). The severity and character of deficits did not vary significantly between children with cardioembolic and non-cardioembolic stroke. In binary logistic regression analysis, higher PedNIHSS scores were independently associated with lower odds of normal outcome (OR 0.819; 95% CI, 0.728–0.922; *P* < .001), whereas cardioembolic aetiology was not independently associated with outcome (*P* = .984).

**Table 3:** Outcome in children with cardioembolic and non-cardioembolic arterial ischaemic stroke.

	All cardioembolic (*n* = 31)	Non-cardio embolic (*n* = 94) ^a^	*P*-value ^b^	Definite cardioembolic (*n* = 15)	*P*-value ^c^
	*n* (%)	*n* (%)		*n* (%)	
**PSOM severity classification system**					
** Normal**	16 (51.6)	53 (56.4)	.681	4 (26.7)	.05
**Mild**	7 (22.6)	19 (20.2)	.801	6 (40.0)	.105
**Moderate**	4 (12.9)	12 (12.8)	1	2 (13.3)	1
**Severe**	4 (12.9)	10 (10.6)	.747	3 (20.0)	.384
**Severe deficits**	3 (9.7)	5 (5.3)	.408	2 (13.3)	.247
** Dead < 2 years from AIS**	1 (3.2)	5 (5.3)	1	1 (6.7)	1
**Character of deficits**					
**Sensorimotor deficits**	13 (41.9)	44 (46.8)	.682	10 (66.7)	.175
**Language—production**	5 (16.1)	14 (14.9)	1	2 (13.3)	1
**Language—comprehension**	1 (3.2)	2 (2.1)	1	1 (6.7)	.361
**Cognitive or behavioural deficits**	8 (25.8)	23 (24.5)	1	3 (20.0)	1

Abbreviations: AIS, arterial ischaemic stroke; PSOM, paediatric stroke outcome measure.

^a^Missing data on clinical outcomes in one child with AIS.

^b^Fisher’s exact test: cardioembolic vs non-cardioembolic.

^c^Fisher’s exact test: definite cardioembolic vs non-cardioembolic.

When limiting the analysis to children categorised as definite cardioembolic stroke, normal clinical outcomes were significantly less frequent in the cardioembolic than in the non-cardioembolic group (26.7% vs 56.4%, *P* = .050) (Table 3). In binary logistic regression analysis, higher PedNIHSS scores remained independently associated with lower odds of normal outcome (OR 0.846; 95% CI, 0.741–0.965; *P* = .013), while definite cardioembolic stroke was not independently associated with outcome (OR 0.321; 95% CI, 0.087–1.179; *P* = .087).

## Discussion

In this nationwide, retrospective population-based study, we examined the incidence rate and outcomes of paediatric cardioembolic stroke. Cardioembolic stroke was identified in 31 of 126 children, corresponding to an incidence rate of 0.28 per 100,000 person-years. The most common underlying conditions were CHDs present in more than 80% of children with cardioembolic stroke. Neurological deficits were observed in about half of children with AIS and clinical outcome was similar between children with cardioembolic and non-cardioembolic stroke.

While clinical outcomes were overall similar between groups, univariable analysis demonstrated that children with definite cardioembolic stroke were less likely to achieve a normal functional outcome than children with non-cardioembolic AIS (26.7% vs 56.4%, *P* = .050). Previous paediatric stroke studies have suggested that adverse outcomes in children with cardiac disease may reflect the neurodevelopmental vulnerability associated with hypoxemia and chronic cerebral hypoperfusion.^3^ However, in this study, multivariable analysis showed that higher PedNIHSS scores were independently associated with lower odds of normal outcome (OR 0.819; 95% CI, 0.728–0.922; *P* < .001), while cardioembolic aetiology was not (*P* = .984). These findings indicate that functional outcome after paediatric AIS is primarily determined by stroke severity rather than aetiology.

In adults, cardioembolic stroke has been associated with a higher risk of LVOs compared to other AIS etiologies.^18^ We observed a similar, although non-significant, trend towards a higher frequency of LVOs in children with cardioembolic AIS compared to those with other aetiologies (41.2% vs 18.5%; *P* = .06). However, given the proportion of children who underwent angiography (66%), this finding requires confirmation in other paediatric AIS cohorts.

The incidence of paediatric cardioembolic stroke was lower in our study (0.28 per 100,000 person-years) than in a previous retrospective cohort study from Canada (0.39 per 100,000 person-years).^19^ Several factors may contribute to this difference, including variations in study designs and methodologies as well as temporal differences in AIS prevention among children with cardiac disease. The Canadian study was conducted more than 2 decades ago. Since then, primary AIS prevention may have improved alongside increased use of antithrombotic therapy in children.^20^

Like previous studies, we observed a high frequency of CHDs in children with AIS. In population-based case–control studies, the presence of CHD has been associated with increased risk of neurodevelopmental disorders.^21^ While this finding has been suggested to reflect a common genetic aetiology, the association may also reflect increased stroke risk in children with heart disease. Two recent studies from Sweden demonstrated a significantly higher prevalence of autism and Attention Deficit Hyperactivity Disorder (ADHD) in children with AIS compared to healthy controls.^22^^,^^23^ In consistency with these findings, we observed a high frequency of cognitive and behavioural deficits in children with AIS (cardioembolic 25.8%; non-cardioembolic 24.5%).

Patent foramen ovale accounted 42% of cardioembolic AIS events in our cohort. While PFO is frequently reported as a possible AIS risk factor,^24^^,^^25^ its high prevalence in otherwise healthy children complicates interpretation of its impact on stroke risk and related treatment decisions. In our study, PFO closure was performed in no children, though considered in one (data not reported). To guide decisions on PFO closure, the Risk of Paradoxical Embolism score has been used in adults^26^; however, the score’s applicability in children is limited by age-related differences in AIS risk factors.^27^ As PFO closure has been shown to reduce AIS recurrence rates in selected adults,^28^ further research is needed to inform the selection of children for the procedure.

We observed that, neurological deficits were present in about half of children with AIS. A previous retrospective cohort study from Spain^3^ found high rates of neurological impairments following paediatric stroke as our study. They reported unfavourable outcomes in approximately 70% of patients. Similar to our findings, the neurological impairments were predominant involvement of sensorimotor and cognitive-behavioural domains.

In our study, 9 patients underwent cardiac procedures. Three out of 9 patients were on preventive antithrombotic treatment. None of the children underwent pre-operational screening for intra-cardiac thrombi. According to a scientific statement by the American Heart Association and American Stroke Association,^29^ primary prevention may be achieved in adults by screening for intra-cardiac thrombi prior to an operation, and if present postponing operation, and start of antithrombotic therapy.^29^ A similar approach may reduce AIS risk in children with cardiac disease.

### Strength and limitations

The strengths of this study include the nationwide, population-based design and validation of possible AIS events. The Danish healthcare system is universal and tax-supported limiting the influence of financial capacity on referral and treatment. Our long study period from 2013 to 2020 further strengthens the study.

Several limitations should be acknowledged. The retrospective design is inherently subject to missing or incomplete data and potential misclassification of stroke aetiology and outcomes. For example, baseline functional status, including mRS, was not systematically documented in the medical records and therefore could not be included in the analyses. The follow-up period in this study was limited to 2 years, a longer follow-up period could contribute to a better understanding of the long-term outcomes. Finally, the relatively small sample sizes in certain subgroups, particularly children with definite cardioembolic stroke, may limit statistical power to detect subtle differences in neurological outcomes.

## Conclusion

In this nationwide study, cardioembolic stroke accounted for nearly one-quarter of paediatric AIS events. Among children undergoing angiography, LVOs were present in 23% and the frequency seemed higher in children with cardioembolic stroke than non-cardioembolic stroke. While overall outcomes were similar between cardioembolic and non-cardioembolic stroke, children classified with definite cardioembolic disease were significantly less likely to achieve normal functional outcomes. Our findings highlight the need for improved primary prevention and acute management of cardioembolic stroke in children.

## Data Availability

The data that support the findings of this study are available from the corresponding author upon reasonable request.
